# Prognostic role of nutritional status in elderly patients hospitalized for COVID-19: a monocentric study

**DOI:** 10.1007/s40520-020-01727-5

**Published:** 2020-10-08

**Authors:** Guerino Recinella, Giovanni Marasco, Giovanni Serafini, Lorenzo Maestri, Giampaolo Bianchi, Paola Forti, Marco Zoli

**Affiliations:** 1 Unit of Internal Medicine, Department of Medical and Surgical Sciences, S. Orsola- Malpighi University Hospital, University of Bologna, Via Pietro Albertoni 15, 40138 Bologna, Italy; 2 Department of Medical and Surgical Sciences, S. Orsola-Malpighi Hospital, University of Bologna, Via Pietro Albertoni 15, 40138 Bologna, Italy

**Keywords:** COVID-19, Elderly, Nutrition, Geriatric nutritional risk index

## Abstract

**Background:**

Symptomatic severe acute respiratory syndrome-coronavirus-2 (SARS-CoV-2) infection incidence is higher in the elderly patients. Pre-existing geriatric conditions such as comorbidity and frailty seem related to worse hospital outcomes.

**Aims:**

To assess the role of nutritional status as an independent prognostic factor for in-hospital death in elderly patients.

**Methods:**

Consecutive elderly patients (age > 65 years) hospitalized for novel coronavirus disease (COVID-19) were enrolled. Demographics, laboratory and comorbidity data were collected. Nutritional status was evaluated using the Geriatric Nutritional Risk Index (GNRI). Uni- and multivariate Cox regression analyses to evaluate predictors for in-hospital death were performed.

**Results:**

One hundred and nine hospitalized elderly patients (54 male) were consecutively enrolled. At univariate analysis, age (HR 1.045 [CI 1.008–1.082]), cognitive impairment (HR 1.949 [CI 1.045–3.364]), C-reactive protein (HR 1.004 [CI 1.011–1.078]), lactate dehydrogenases (HR 1.003 [CI 1.001–1.004]) and GNRI moderate–severe risk category (HR 8.571 [CI 1.096–67.031]) were risk factors for in-hospital death, while albumin (HR 0.809 [CI 0.822–0.964]), PaO_2_/FiO_2_ ratio (HR 0.996 [CI 0.993–0.999]) and body mass index (HR 0.875 [CI 0.782–0.979]) were protective factors. Kaplan–Meier survival curves showed a significative higher survival in patients without GNRI moderate or severe risk category (*p* = 0.0013).

At multivariate analysis, PaO_2_/FiO_2_ ratio (HR 0.993 [CI 0.987–0.999], *p* = 0.046) and GNRI moderate–severe risk category (HR 9.285 [1.183–72.879], *p* = 0.034) were independently associated with in-hospital death.

**Conclusion:**

Nutritional status assessed by GNRI is a significative predictor of survival in elderly patients hospitalized for COVID-19. The association between GNRI and PaO_2_/FiO_2_ ratio is a good prognostic model these patients.

## Introduction

Since the beginning of the novel coronavirus disease (COVID-19) emergency in Italy, more than 200,000 infections and more than 30,000 deaths have been documented.

Although most patients suffer from a mild illness, a relatively high percentage of patients need to be hospitalized so the pandemic has put hospital systems under strain [1, 2]. The majority of hospitalized patients are elderly [3].

These patients undergo to higher mortality mainly due to their frailty, the presence of comorbidity and high degree of disability [4–6]. However, only a few studies evaluated the epidemiological characteristics and the predictive factors of unfavourable outcomes in elderly patients with COVID-19 [7, 8].

The alterations of the immune system of the elderly patients may have an important prognostic role. Indeed, the remodelling of the immune response observed among the elderly could explain the increased prevalence of more aggressive clinical manifestations of COVID-19 in these patients [9]. In particular, the state of chronic inflammation when not under control loses its defensive role and turns into a damaging state to the whole organism; the practical consequence is that inflamm-aging predicts frailty, and is associated with higher mortality.

Malnutrition is another possible explanation for the worse outcomes of elderly patients symptomatic COVID-19. This geriatric syndrome has a multifactorial aetiology and is strongly related to frailty and negative hospital outcomes in patients admitted with acute illnesses [10].

Although a prognostic role of nutritional status in elderly patients with COVID-19 has been hypothesized [11–13], to our knowledge, no study has assessed the prognostic value of nutritional status in elderly patients hospitalized for COVID-19.

We aimed to assess the prognostic role of nutritional status for in-hospital death of elderly patients hospitalized for COVID-19.

## Methods

The study included 109 patients consecutively admitted to two COVID-19 units of Sant’Orsola-Malpighi University Hospital in Bologna between 30 March 30th and May 15th 2020. Inclusion criteria were (1) age ≥ 65; (2) diagnosis of COVID-19 based on the detection of severe acute respiratory syndrome-coronavirus-2 (SARS-CoV-2) on reverse transcriptase polymerase chain reaction (RT-PCR) from the nasopharyngeal swab. Exclusion criteria were the presence of terminal neoplasia and the exclusively clinical and radiological diagnosis of COVID-19 without laboratory confirmation. Demographics, past medical history, and clinical and laboratory data on admission were recorded by patient’s medical record. Comorbidity was assessed using the Charlson Comorbidity Index (CCI) [14]. Clinical and laboratory indicators of severity for SARS-COV-2 infection included lymphocytes, lactate dehydrogenases (LDH), C-reactive protein, D-dimer, and partial pressure of oxygen/fraction of inspired oxygen ratio (PaO_2_/FiO_2_).

Nutritional status was evaluated using the Geriatric Nutritional Risk Index (GNRI) within 48 h of admission. The GNRI is a simple nutritional screening tool used to evaluate nutrition-related risk in surgical and medical patients [15, 16]. The index was calculated as follows: GNRI = 1.489 × serum albumin (g/L) + 41.7 × present weight/ideal weight (kg). Ideal body weight was derived using the equations of Lorentz [17]: ideal weight for men = 0.75 × height (cm) − 62.5, ideal weight for women = 0.60 × height (cm) − 40. According with previous study [18], three categories were identified: no risk (GNRI > 98), low risk (GRNI 92–98) and severe–moderate risk (GNRI < 92).

Patients’ follow-up started at admission and was carried out until hospital discharge or death. The evaluated outcome was in-hospital death. The study was conducted according to the declaration of Helsinki's ethical principles for medical research involving human subjects. Informed consent was obtained from each patient (or from patient’s relatives if the subject was disabled). The protocol was reviewed and approved by the Ethics Committee of the S. Orsola University Hospital (Protocol number 512/2020/Oss/AOUBO).

### Statistical analysis

Data were reported as median with interquartile range (IQR) for continuous variables and numbers and percentages for categorical variables. Comparisons between patients grouped by vital status at discharge were analyzed by Fischer, Chi-square or Mann–Whitney tests when appropriate. Subsequently, the same variables were tested as independent variables associated with in-hospital death. First, several univariate Cox regression analyses were performed considering statistically significant those variables with *p* value less than 0.1. Subsequently, only the variables significantly associated with the in-hospital death in univariate analyses were entered into a multivariate model, excluding collinear variables. Finally, the best multivariate model was identified, adopting a backward elimination procedure. The estimated hazard ratios (HR) with their 95% confidence intervals (95% CI) were calculated; *p* values less than 0.05 were considered statistically significant. The results obtained from multivariate analysis, in the presence of two or more co-variates influencing the risk, were translated in graphic form through the use of nomograms for Cox regression analyses.

A Kaplan–Meier survival curve to estimate the survival according to GNRI categories (no risk/low risk vs moderate risk/severe risk) was constructed; the statistical significance of differences between GNRI categories was tested with the log-rank test. Statistical analyses were performed using Stata/SE (Version 13.0; Stata Corp, Texas, United States of America) for Windows.

## Results

One-hundred and nine patients (109, 54 male 51.4%) were consecutively included in our study. The median age was 83 years (76–91.5). During a median follow-up of 11 (8–15) days, 43 (39.4%) patients died. Most of the patients enrolled reported other underlying comorbidities: arterial hypertension (75.2%), cognitive impairment (44%) and atrial fibrillation (29.4%) were the most common. During hospitalization, six patients (5.5%) were transferred to the intensive care unit (ICU). None (0%) of patients transferred to ICU had moderate to severe GNRI, while 67 (65%) patients in the group “no transfer to ICU” had moderate to severe GNRI.

### Demographics and clinical status

Table 1 shows characteristics of patients grouped by vital status at discharge. In-hospital death was associated with higher age, longer hospital stay, cognitive impairment, and clinical and laboratory indicators of more severe disease (higher prevalence of dyspnea on admission, higher C-reactive protein and LDH, lower PaO_2_/FiO_2_).

**Table 1:** Differences in demographic, clinical and laboratory findings between group of patients experiencing in-hospital death and not

	No in-hospital death (*n* = 66), *n* (%) or median [IQR]	In-hospital death (*n* = 43), *n* (%) or median [IQR]	*p *value
Age	79 [74–92]	85.5 [79–86.7]	0.007
Male	32 (48.5)	22 (51.2)	0.785
Length of hospital stay, day	8 [7–15]	11 [8–15]	0.010
Comorbidity			
Charlson Comorbidity Index	4 [3–6]	4.5 [2.2–8.7]	0.817
Cognitive impairment	21 (31.8)	27 (62.8)	0.001
Previous stroke	7 (10.6)	10 (23.3)	0.075
Arterial hypertension	49 (74.2)	43 (76.7)	0.767
Diabetes	16 (24.2)	8 (18.6)	0.488
Atrial fibrillation	19 (28.8)	13 (30.2)	0.871
Coronary heart disease	9 (13.6)	6 (14)	0.963
Chronic heart failure	9 (13.6)	8 (18.6)	0.485
COPD	12 (18.2)	12 (27.9)	0.231
CKD	15 (22.7)	8 (18.6)	0.606
Symptoms			
Fever	43 (65.2)	25 (58.1)	0.460
Dyspnea	20 (30.3)	30 (69.8)	< 0.001
Cough	24 (36.4)	14 (32.6)	0.684
Asthenia	21 (31.8)	14 (32.5)	0.653
Laboratory features and nutritional parameters			
PaO_2_/FiO_2_	319 [276–347]	254 [175–286]	0.006
Lymphocytes, 10^9^/L	1.01 [0.81–1.32]	0.72 [0.45–1.12]	0.071
C-reactive protein, mg/dL	6.35 [1.09–11.32]	13.38 [4.24–19.14]	0.003
LDH, U/L	222 [188–310]	266 [197–381]	0.012
D-dimer, μg/mL	1.12 [0.5–2.49]	1.99 [0.79–3.79]	0.140
GRF, mL/min	67 [33–81]	46.5 [39.7–78.2]	0.281
Albumin, g/L	31.6 [28.3.8–35.3]	26.3 [23.9–30.7]	< 0.001
Weight, kg	73 [63–80]	55 [45–73.5]	0.001
BMI, kg/m^2^	25.7 [22.5–28.5]	20.3 [16–23.9]	0.002
GNRI	95 [88–103]	82 [69–87.5]	< 0.001
No risk	26 (39.4)	4 (9.3)	0.058
Low risk	12 (18.2)	0 (0)	0.124
Moderate–severe risk	28 (42.4)	39 (90.7)	0.004

*n* = numbers, *IQR* interquartile range, *BMI* body mass index, *COPD* chronic obstructive pulmonary disease, *CKD* chronic kidney disease, *FiO*_*2*_ fraction of inspired oxygen, *GNRI* Geriatric Nutrition Risk Index, *GRF* glomerular filtration rate, *LDH* lactate dehydrogenases, *PaO*_*2*_ partial pressure of oxygen

### Assessment of nutritional status

Considering the nutritional parameters, lower values of body weight (*p* = 0.001), BMI (*p* = 0.002) and albumin (*p* < 0.001) were found in patients experiencing in-hospital death. On the other hand, higher values of GNRI were found in surviving patients (*p* < 0.001) and a higher prevalence of GNRI moderate–severe risk category was found in the in-hospital death group (*p* = 0.004). Nutritional parameters are summarized in Table 1.

### Independent in-hospital death predictors

Among all variables evaluated in the univariate Cox regression analysis (Table 2), age, cognitive impairment, C-reactive protein, LDH, and GNRI moderate-severe risk category (HR 8.571 [CI 1.096–67.031] *p* = 0.041) were associated with in-hospital death, while albumin, body mass index, and PaO_2_/FiO_2_ ratio showed a protective role.

**Table 2:** Univariate and multivariate analyses for independent variables associated with in-hospital death

	Univariate		Multivariate	
	HR (95% CI)	*p* value	HR (95% CI)	*p* value
Age	1.045 (1.008–1.082)	0.014		
Sex	1.109 (0.609–2.018)	0.734		
Charlson Comorbidity Index	0.963 (0.835–1.111)	0.612		
Cognitive impairment	1.949 (1.045–3.364)	0.036		
Coronary heart disease	1.018 (0.428–2.418)	0.967		
Atrial fibrillation	1.111 (0.579–2.132)	0.750		
Arterial hypertension	1.006 (0.495–2.047)	0.985		
Diabetes	0.751 (0.347–1.622)	0.466		
Previous stroke	1.431 (0.704–2.908)	0.322		
Chronic heart failure	1.024 (0.472–2.223)	0.951		
CKD	1.251 (0.572–2.732)	0.574		
COPD	1.434 (0.735–2.799)	0.291		
Lymphocytes	1.138 (0.834–1.554)	0.415		
C-reactive protein	1.044 (1.011–1.078)	0.008		
LDH	1.003 (1.001–1.004)	< 0.001		
D-dimer	1.019 (0.976–1.065)	0.383		
GRF	0.994 (0.983–1.004)	0.286		
Albumin	0.890 (0.822–0.964)	0.004		
PaO_2_/FiO_2_	0.996 (0.993–0.999)	0.008	0.993 (0.987–0.999)	0.046
BMI	0.875 (0.782–0.979)	0.020		
GNRI (moderate–severe risk)	8.571 (1.096–67.031)	0.041	9.285 (1.183–72.879)	0.034

*BMI* body mass index, *CKD* chronic kidney disease, *COPD* chronic obstructive pulmonary disease, *FiO*_*2*_ fraction of inspired oxygen, *GFR* glomerular filtration rate, *GNRI* Geriatric Nutrition Risk Index, *LDH* lactate dehydrogenases, *PaO*_*2*_ partial pressure of oxygen

**Fig. 1:**
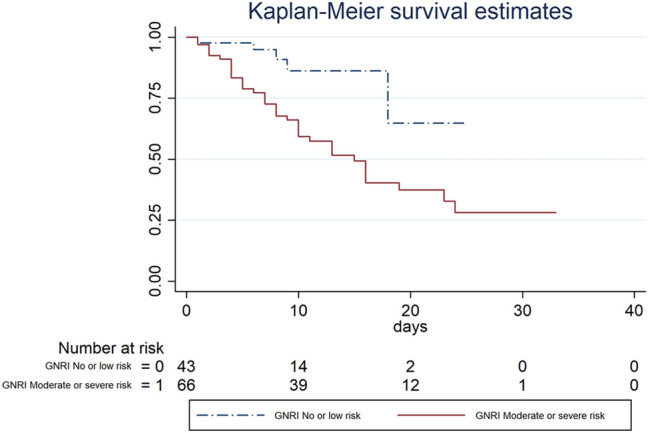
Kaplan-Meier survival curves according to the GNRI groups: no or low risk group versus moderate or high risk group

Kaplan–Meier survival curves according to the GNRI were estimated (Fig. 1). A higher statistically significant survival was found in the group without risk or low risk according to GNRI, compared to those with moderate risk or severe risk (*p* = 0.003).

**Fig. 2:**
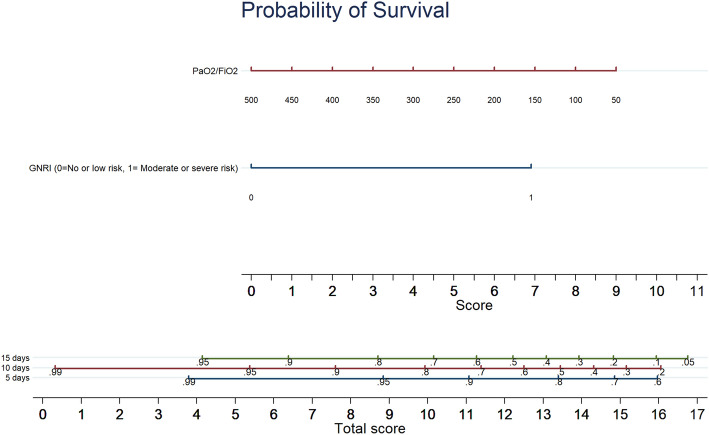
A nomogram with GNRI values and PaO2/FiO2 ratio showing the different probability of survival at 7, 14, and 21 days after hospital admission

Multivariate Cox regression analysis showed that GNRI moderate-severe risk category (HR 9.285 [1.183–72.879], *p* = 0.034) and PaO_2_/FiO_2_ ratio (HR 0.993 [CI 0.987–0.999], *p* = 0.046) were the only independent predictors of in-hospital death. The results of the multivariate Cox regression analysis were graphically reported in a nomogram showing the different probability of survival at 7, 14, and 21 days after hospital admission (Fig. 2).

## Discussion

The main result of our study is that impaired nutritional status, assessed by the GNRI, together with the PaO_2_/FiO_2_ ratio, is an independent predictor of in-hospital mortality in elderly patients with symptomatic SARS-CoV-2 infection.

To our knowledge, this is the first study reporting data on the predictive ability of nutritional scales in elderly patients hospitalized for SARS-CoV-2 infection.

We assessed nutritional status using GNRI which is a score already validated in the literature both in surgical and clinical settings [15, 16, 19]. Our study shows that GNRI is also a possible prognostic tool for mortality of elderly patients with COVID-19. In fact, an increased risk of in-hospital mortality was present in the GNRI moderate–severe risk group with a ninefold increase in risk in the multivariate model (HR 9.285 [1.183–72.879], *p* = 0.034). Thus, the pre-hospital status of the elderly patient, not only in terms of comorbidity, but also in terms of nutritional parameters, represents one of the main prognostic factors in patients hospitalized for COVID-19.

In line with these results, our elderly population showed higher albuminemia values in the group of patients not experiencing in-hospital death (*p* < 0.001); this protective relationship was also confirmed by the univariate analysis (HR 0.809 [CI 0.822–0.964]). In addition, a peculiar result of our study concerns BMI; previous studies in not age-selected population reported that an higher BMI was associated with unfavourable outcomes in subjects with COVID-19, in particular higher risk of hospitalization, risk of ICU transfer and length of ICU stay, and finally of death [20]. Surprisingly, in our study, a higher values of BMI showed a protective role; this may probably be due to the study design, including only subjects with age over 65 years. Indeed, in the elderly population, a higher BMI value could be translated in a better nutritional status [21], thus explaining our findings.

Our observations agree with previous evidence that malnutrition is associated with an increased mortality from both infections and chronic diseases [21, 22]. There are several reasons for this association, including the close association between malnutrition and immune system deficit [10, 23].

We, therefore, believe that maximum attention should be paid to COVID-19 elderly patient with malnutrition and where possible start quickly a supplementary feeding.

Another factor independently associated with intra-hospital mortality was the PaO_2_/FiO_2_ ratio. This finding agrees with previous studies in adult populations [24, 25] and may be due to a greater pulmonary involvement in patients with low values of this ratio.

Another novelty of this study is represented by the use of nomograms that were elaborated for evaluating the probability of survival of elderly patients hospitalized for COVID19; the nomogram provided represent an easy-handling tool for rapidly calculating the probability of survival for a single patient in the era of tailored medicine.

Only a few previous researches described the clinical and epidemiological characteristics of elderly patients hospitalized for SARS-CoV-2 infection [5, 6, 8, 26]. Similar to our study, most patients were characterized by a high number of chronic comorbid condition and pre-existent functional disability.

In our study, we did not assess cognitive impairment on standardized scales, but data were collected according to the medical history. Nevertheless, our results show that cognitive impairment was highly prevalent among patients who died before discharge; however, multivariable analyses did not confirm cognitive impairment as an independent predictor. In other studies assessing the predictive value of cognitive impairment in COVID-19 patients, it has been reported to be a predictive factor for unfavourable outcomes [26, 27]. Several reasons may explain this association and why it disappeared in favor of GNRI and PaO_2_/FiO_2_ ratio. First, patients with cognitive impairment have poor compliance with prescriptions [28] such as oxygen therapy; in addition, cognitive impairment is closely related to sarcopenia and malnutrition, which in turn is associated with increased adverse outcomes [29].

In our elderly population, the most frequent COVID-19 clinical symptoms were fever, cough and dyspnea. Notably, dyspnea prevalence was significantly higher in patients experiencing in-hospital death. This suggests that the greater the respiratory involvement, the worse the outcome of COVID-19 patients.

Our study has some limitations. First, due to the monocentric design, the enrolled population may be not representative of the elderly patients hospitalized for COVID-19. A second limitation is the lack of data about other relevant parameters of body fat and muscle mass (e.g. waist circumference, muscle thickness). However, given the dramatic nature of the situation, it was difficult to collect complex nutritional parameters even to minimise the risk for health workers.

In addition, our multivariate analysis may be affected by an overfitting variable bias since we reported only 43 death; moreover, our predictive model still needs an internal and external validation.

However, our study has also several strengths. This is the first report including nutritional parameters as a prognostic factors in elderly patients hospitalized for COVID-19; in addition, the assessment of nutritional status has been carried out using a simple, rapid and effective score especially in high infectious risk contexts where complex measurements cannot be carried out. Finally, we provided a nomogram able to personalize the individual probability of survival of each geriatric patient hospitalized for COVID-19. Future larger and multicentric studies are needed to validate our results on the prognostic value of GNRI in predicting in-hospital death and to further explore the role of other nutritional parameters in elderly patients hospitalized for COVID-19.

## Conclusion

Nutritional status assessed by GNRI is an independent prognostic factor for in-hospital mortality. The association of GNRI with a respiratory parameter (PaO2/FiO2) provides a comprehensive prognostic tool for predicting adverse outcomes in elderly COVID-19 patients.
